# Approaches to Identifying STEM Talent in K-12 Gifted Education: A Systematic Review

**DOI:** 10.3390/jintelligence14080153

**Published:** 2026-07-24

**Authors:** Jiaqi Tang, Huichao Bi, Jun Wei

**Affiliations:** School of Education, Tsinghua University, Beijing 100084, China; tangjq23@mails.tsinghua.edu.cn

**Keywords:** STEM education, gifted learners, identification, K-12 STEM talent

## Abstract

In the era of digital transformation and artificial intelligence, strengthening STEM education is indispensable to technological innovation and social progress. However, the scarcity of effective strategies and empirical evidence for the early identification of STEM-gifted learners constitutes a critical research gap. This study employed a systematic review approach to synthesize existing identification strategies for K-12 STEM-talent learners. Following the PRISMA framework, 26 peer-reviewed empirical and review articles were retrieved and analyzed from nine databases. Findings revealed that the majority of studies originated in the United States, peaked in 2018, and predominantly comprised empirical designs targeting high school students. Identification methods demonstrated an observed shift from holistic to more individualized approaches, encompassing intelligence and spatial ability testing, academic achievement measures, creativity and problem-solving assessments, portfolio evaluations, subject-specific competitions, and teacher or parent recommendations. Cognitive reasoning, creativity, spatial ability, and problem-solving emerged as core indicators, whereas socioeconomic status, cultural background, teacher and parental support, and gender expectations significantly influenced identification processes and talent development. Future research should empirically validate existing identification framework, account for cross-cultural variability, and examine the longitudinal efficacy of emerging artificial intelligence applications. This review synthesizes existing scholarship on K-12 STEM talent identification and delivers evidence-based recommendations to refine identification and assessment frameworks across diverse educational contexts.

## 1. Introduction

The rapid advancement of scientific discovery and artificial intelligence has reconfigured global economic and social landscapes, fueling the demand for STEM talent capable of driving innovation and technological leadership. Within this milieu, STEM expertise has emerged as a principal driver of economic competitiveness and social progress (Omar, 2024). To secure a vanguard position in global innovation, many nations have substantially increased investments in K-12 STEM education through policy initiatives and financial commitments (Athanasia & Cota, 2022; Tanenbaum, 2016; Zhan et al., 2022). Landmark examples include the United States’ K-12 Science Education Framework (National Research Council, 2012), the United Kingdom’s Science and Technology Framework (Department for Science, Innovation and Technology, 2023), and Finland’s LUMA Centre Finland (Vartiainen et al., 2016), all reflecting a shared commitment to equipping younger generations with advanced competencies for the 21st century.

Against this backdrop, cultivating high-quality, multidisciplinary STEM talent has emerged as both an educational priority and a societal imperative. Gifted education plays a pivotal role by identifying students with exceptional potential during K-12 education (approximately ages 5–18) and providing them with specialized, tailored learning opportunities (Olszewski-Kubilius, 2023). The Talent Development Framework (Olszewski-Kubilius et al., 2018) underscores that early identification is critical to maximizing long-term outcomes. Yet, despite decades of practice, effectively identifying STEM talent remains a persistent challenge. Existing assessment approaches, while valuable, often fall short in precision, reliability, and responsiveness to demographic diversity, resulting in inequitable recognition and support (Johnsen & van Tassek-Baska, 2022). Furthermore, the 21st Century Skills Framework (P21 Framework Definitions, 2009; Guo & Woulfin, 2016) and the Framework for K-12 Science Education (National Research Council, 2012) call for broader, domain-sensitive identification strategies that align with the multifaceted nature of STEM expertise.

Although prior research has explored gender disparities (Heilbronner, 2013), technology integration (Gül & Ayik, 2024), higher-order thinking skills (Haryadi et al., 2021), spatial ability development (Yoon & Mann, 2017), and contextual factors such as rurality and socioeconomic disadvantage (Morris et al., 2021; Ihrig et al., 2018), systematic syntheses of K-12 STEM talent identification strategies remain scarce. Existing review studies (e.g., Gül & Ayik, 2024; Herce-Palomares et al., 2022) tend to provide broad overviews rather than in-depth analyses of identification methodologies. This gap limits the translation of research into practice and underscores the need for a theoretically grounded, domain-specific synthesis.

### 1.1. Historical Overview of Talent Identification

The evolution of gifted education and its identification methods paralleled societal values, scientific insights, and educational goals, undergoing significant transformations from the 19th century to the present day. This historical trajectory spanned from the 19th-century hereditarian theories, with British research led by Francis Galton’s “Hereditary Genius” (Galton, 1869) emphasizing genetic predispositions, to the diverse assessment models of the 20th century, such as Howard Gardner’s theory of multiple intelligences (Gardner, 1983), and the 21st century’s focus on innovation and personalized learning, as highlighted by the work of David Dai (Dai, 2016). The field has broadened its understanding of “genius,” influencing global educational reform and talent identification strategies.

In the 19th century, initial theories of giftedness were rooted in genetic explanations. Other countries focused more on educational and philosophical discussions, lacking a formal framework for studying geniuses. By the early 20th century, Western countries had begun to adopt intelligence testing as a core identification tool, supplemented by teacher recommendations and academic achievements. France implemented the Binet–Simon Intelligence Test (Binet & Simon, 1905), while the U.S. and U.K. utilized the Stanford–Binet Intelligence Test (Terman, 1916), and Germany relied on academic performance and teacher recommendations (Dienstbach, 1925).

By the mid-20th century, global talent identification methods had expanded to include intelligence tests, creativity assessments, achievement tests, and teacher recommendations (Gallagher, 1964; Kerr, 1991). Although the IQ test remained prevalent, creativity assessments and multidimensional evaluations were integrated as diversified identification approaches. In the U.S., the Wechsler Intelligence Scale (Wechsler, 1949) and Stanford–Binet Intelligence Test (Terman, 1916) continued to be widely employed, with the U.K. adopting Raven’s Progressive Matrices (Raven, 1936), and France retaining the Binet–Simon Test (Binet & Simon, 1905). In addition, the U.S. incorporated the Torrance Tests of Creative Thinking (Torrance, 1966), and academic testing gained prominence in various countries, exemplified by the SAT and ACT in the U.S. (Snyder et al., 2013), the eleven-plus in the U.K. (Britannica, 1998), the National Center Test in Japan (Watanabe, 2013), and the Abitur exam in Germany (Laycock, 1979).

In the 21st century, gifted education continues to evolve, expanding STEM talent identification to emphasize innovation, problem-solving, and personalized learning experiences. In the U.S., influenced by the Marland Report (Marland, 1972), project-based learning fosters innovative thinking in gifted students (Lucas Education Research, 2021). The U.K., in addition to the GCSE and A-Level exams, has implemented project-based learning, problem-solving, and interdisciplinary methods. Finland emphasizes personalized learning and problem-solving abilities (Makkonen et al., 2021), while Singapore’s Gifted Education Programme offers tailored curricula (Ministry of Education, Singapore, 2026), and Australia has introduced individualized education plans for gifted learners (Gross, 2003), later reinforced by updated state-level high-potential and gifted education policies (e.g., NSW Department of Education, 2021). Moreover, Singapore, South Korea, and China encourage students’ innovative abilities through international research partnerships and Olympiads (Tofel-Grehl & Callahan, 2017).

Despite these advances, significant challenges persist. Traditional intelligence testing and hereditarian assumptions often neglect environmental influences, while standardized assessments may fail to capture diverse expressions of talent and creativity. Labeling students as “gifted” risks marginalizing non-traditional abilities and excluding those whose potential remains unrecognized. In response, some education systems have adopted more flexible, inclusive, and personalized approaches, while remaining attentive to the risk of reinforcing structural inequalities and rigid conceptions of talent (Ibata-Arens, 2012). Continued research on domain-specific identification strategies remains essential to advancing equitable and effective gifted education.

### 1.2. Theoretical Perspectives on Giftedness and Talent Identification

The field rested on several foundational frameworks that continued to shape both conceptual understanding and identification practice. Among the most influential were Renzulli’s Three-Ring Conception of Giftedness (Renzulli, 1978, 2005) and Gagné’s Differentiated Model of Giftedness and Talent (DMGT; Gagné, 1985, 2004). Renzulli posited that exceptional performance emerged from the interaction of above-average ability, creativity, and task commitment, shifting the field away from sole reliance on IQ toward holistic, performance-based evidence. Gagné advanced the field further by formally distinguishing between giftedness (natural abilities within the top 10% of age peers) and talent (systematically developed domain expertise), while emphasizing the mediating roles of intrapersonal, environmental, and chance catalysts.

Building on these foundations, WICS model integrated wisdom, intelligence, creativity (Sternberg, 2003), and synthesized thinking as prerequisites for outstanding contribution, later extending the framework to include ethical leadership (Sternberg, 2020). Dai (2021) proposed the Evolving Complexity Theory, framing talent as a time- and context-bound functional attribute that progressed from foundational ability through characteristic adaptation to maximal adaptation oriented toward societal contribution. Subotnik et al. (2021) synthesized these insights into the Talent Development Megamodel, mapping the longitudinal trajectory from latent potential to creative productivity.

Ecological perspectives complemented these developmental accounts. Ziegler’s (2005) Actiotope Model centered on individual action within recursive feedback loops between personal agency and environmental systems, emphasizing that excellence arose from adaptive regulation rather than static traits. In China, Lin and Hu (2012) outlined multistage growth pathways for elite innovators, while Yi (2014) situated creativity within nested cultural spheres. Collectively, these frameworks established that effective identification required a multidimensional, developmentally sensitive, and ecologically grounded approach.

### 1.3. The Necessity of Domain-Specific Talent Identification

These general principles, however, require domain-specific operationalization, particularly in STEM. Domain-specific identification involves deploying targeted methods to assess exceptional potential within circumscribed fields (Subotnik et al., 2019). International exemplars illustrate this trend: South Korea (Han, 2007), and the United States (Arnstein et al., 2023) employ phased, multi-method approaches integrating teacher recommendations, academic aptitude tests, and interviews.

In STEM, effective identification must capture problem-solving agility, scientific creativity, and critical thinking (Hebebci & Usta, 2022), while accounting for socioeconomic background, family environment (Stambaugh & Olszewski-Kubilius, 2020), and gender (Font et al., 2024). Despite these imperatives, systematic syntheses of STEM-specific identification remain sparse relative to domains such as sport (Johnston et al., 2018; Hogan et al., 2021; Sarmento et al., 2018; Mendes et al., 2022). Emerging technologies—particularly artificial intelligence and big data analytics—offer promising avenues for refining identification accuracy, efficiency, and personalization. Future research must adopt theoretically grounded approaches that integrate cognitive, motivational, contextual, and ethical considerations to advance more inclusive and effective STEM talent identification practices.

### 1.4. Present Study

This study undertook a systematic review of identification approaches for gifted learners in K-12 STEM education over the past decade. Aiming to facilitate early identification and targeted support, the findings offered actionable insights for policymakers, school administrators, and educators particularly within educational contexts aligned with those represented in the reviewed literature.

The specific research questions are as follows:

RQ1. What are the characteristics and longitudinal trends in K-12 STEM talent identification research?

RQ2. What are the predominant strategies and protocols emplyed to assess STEM talent within K-12 gifted education framework?

RQ3. What pivotal findings have emerged regarding the factors that influence STEM talent identification?

RQ4. What future research directions should be prioritized to advance scholarly understanding and practical application in STEM talent identification?

## 2. Materials and Methods

### 2.1. Database and Searching Strategy

Given the interdisciplinary nature of STEM talent identification, spanning education, psychology, and cognitive science, a comprehensive search was conducted across nine major bibliographic databases: Web of Science, ERIC, PubMed, PsycINFO, ProQuest, Scopus, EBSCO, SAGE, and Taylor & Francis. The search strategy employed “STEM” and “talent” as core keywords, supplemented by relevant synonyms, to ensure exhaustive coverage of the pertinent literature. Search syntax and indexing conventions were customized for each database to align with its specific search architecture. The initial literature search was executed in August 2024, with an updated search conducted in October 2024 to capture any newly published relevant studies.

To capture the full historical trajectory of how STEM talent identification has been conceptualized, from early intelligence-testing paradigms to contemporary multidimensional approaches, no earliest date limit was imposed. Delimitations regarding topic and population were strictly enforced through the inclusion criteria, restricting the focus to K-12 student populations and studies explicitly addressing STEM talent identification, while excluding general gifted education or routine STEM curriculum interventions. Furthermore, the search was limited to English-language publications to ensure consistent coding and stable inter-rater reliability.

Although systematic reviews are typically excluded from primary source syntheses, we intentionally retained peer-reviewed review articles focused on K-12 STEM talent identification (e.g., Gül & Ayik, 2024; Herce-Palomares et al., 2022). This decision was guided by two methodological rationales. First, as a nascent field, the conceptualization and operationalization of STEM talent identification are often explicated within review articles; retaining these provided critical insight into the theoretical framing of identification constructs. Second, given the scarcity of empirical studies exclusively focused on this topic, excluding reviews would have obscured the field’s cumulative understanding and masked methodological gaps. All retained reviews were subjected to the same coding scheme and quality appraisal (MMAT) as empirical articles to minimize circularity and ensure analytic rigor. Accordingly, this systematic review was conducted and reported in accordance with the PRISMA statement (see Supplementary Material S1).

### 2.2. Data Collection

This study adopted explicit inclusion and exclusion criteria to standardize literature screening and ensure all eligible publications fit the research theme focused on K-12 STEM talent identification. Specifically, eligible articles had to meet all inclusion criteria: first, publications must be peer-reviewed empirical or review articles; second, research subjects must cover K-12 students aged 5 to 18; third, the core research focus must be STEM talent identification, excluding studies concerning general gifted education or routine STEM curriculum teaching; fourth, all included literature must be published in English. Correspondingly, literature that met any of the following conditions was excluded: non-empirical publication types including dissertations, book chapters, and conference abstracts; studies centered on STEM talent cultivation programs instead of talent identification; non-English publications; and research without K-12 student participants.

**Figure 1:**
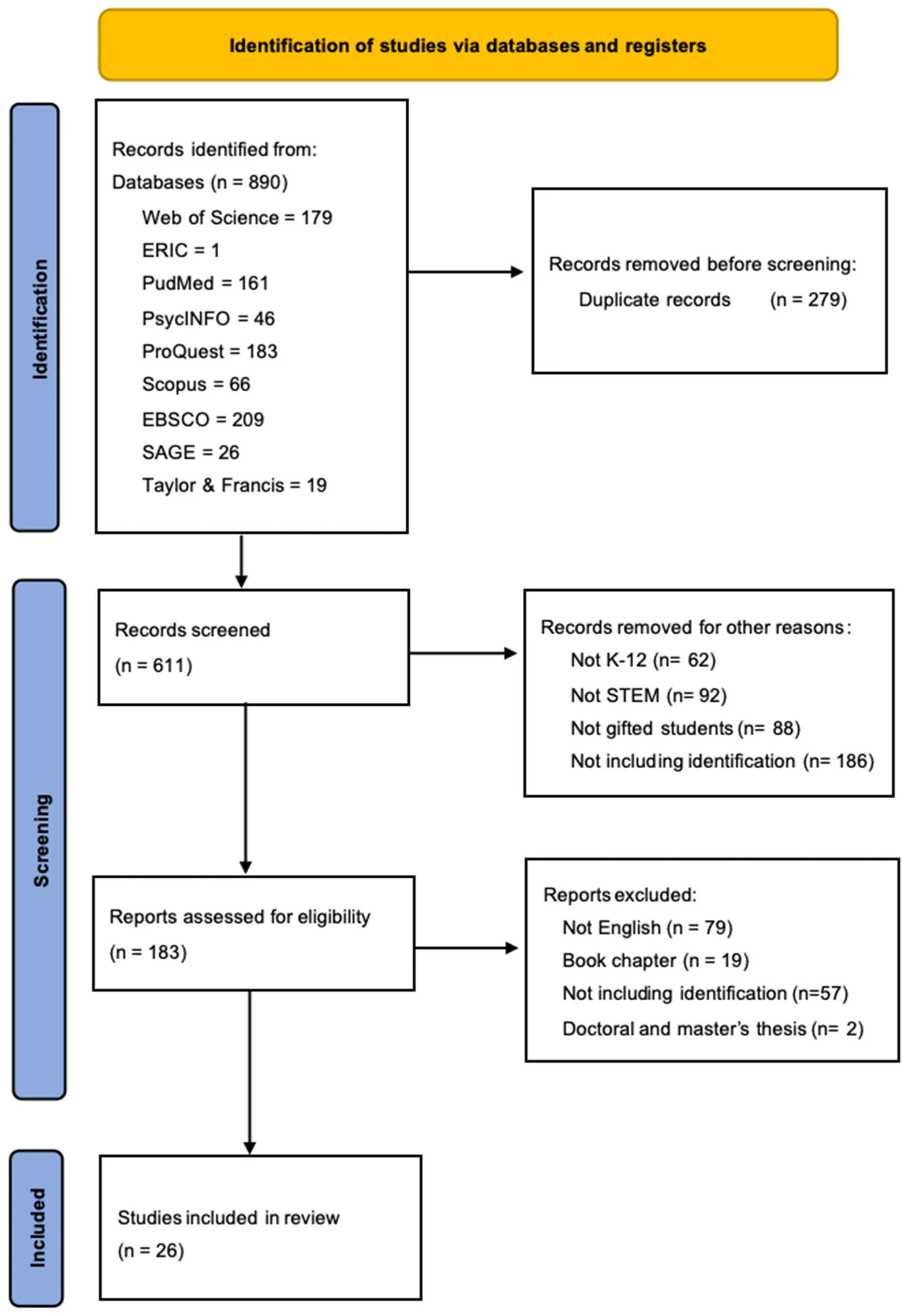
PRISMA Flowchart of Review Process.

As shown in Figure 1, a total of 890 peer-reviewed articles were identified from nine databases, encompassing Web of Science (*n* = 179), ERIC (*n* = 1), PubMed (*n* = 161), PsycINFO (*n* = 46), ProQuest Education Journals (*n* = 183), Scopus (*n* = 66), EBSCO (*n* = 209), SAGE Journals (*n* = 26), and Taylor & Francis Online (*n* = 19). After the elimination of 279 duplicate articles, 611 articles were retained. During the screening of titles and abstracts, 428 articles were excluded for not aligning with the study’s scope, including those not involving K-12 students (*n* = 62), not focusing on STEM (*n* = 92), not pertaining to gifted education (*n* = 88), or not concerning identification (*n* = 186). Following the initial screening, 183 full-text articles were subjected to a thorough review, which led to the exclusion of non-English articles (*n* = 79), book chapters (*n* = 19), articles unrelated to identification (*n* = 57), and doctoral and master’s theses (*n* = 2). Consequently, this systematic review culminated in the inclusion of 26 articles in the final analysis.

### 2.3. Data Analysis

The following information from each screened publication was summarized in Microsoft Excel for quantitative analysis and qualitative comparative synthesis (see Table 1). To ensure inter-rater reliability, two researchers in the field of education were invited to code the literature separately.

The methodological quality of all 26 included studies was independently appraised by two reviewers using the Mixed Methods Appraisal Tool (MMAT, version 2018). Disagreements were resolved through discussion. Quality scores were categorized as high (≥4/5), moderate (3/5), or low (≤2/5) according to MMAT criteria for quantitative, qualitative, and mixed-methods designs. The Cohen’s Kappa coefficient was κ = 0.87 (95% CI [0.82, 0.92]), presenting an almost perfect agreement between the two coders.

## 3. Results

### 3.1. Research Trends on STEM Talent Identification

#### 3.1.1. Overview: Distribution of Studies by Country, Years, Journals, and Research Methods

**Figure 2:**
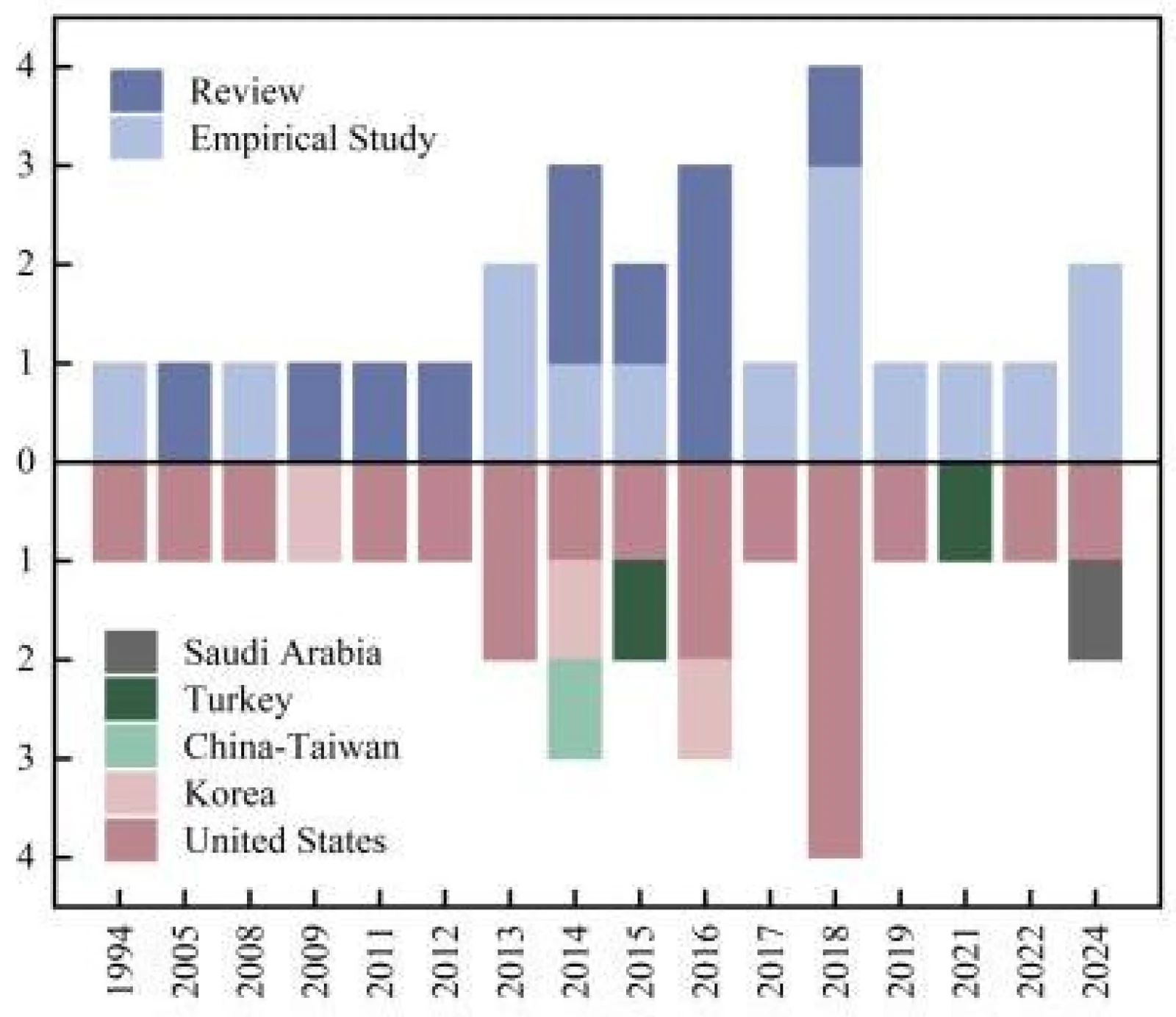
Number of Reviewed Articles on STEM Talent Identification by Year.

This review examined twenty-six articles sourced from nine databases, focusing on the identification of gifted students within K-12 STEM education, with a predominant focus on research conducted in the United States. Over the past three decades, there was a general upward trajectory in the volume of research dedicated to identifying gifted students in K-12 STEM education (see Figure 2). Publications were sparse in the 1990s and early 2000s but began to escalate post-2006. The period between 2011 and 2018 marked a surge in research on talent identification, culminating in a peak in 2018, followed by a slight decline in 2019.

Of the twenty-six articles reviewed, the majority pertained to qualitative studies on talent identification (TI), constituting 55% of the total, with quantitative studies accounting for 32%, and mixed method studies the least frequent, at 13%. Half of the articles (*n* = 13) were published in journals indexed in the Social Sciences Citation Index (SSCI), with the following distribution: Gifted Child Quarterly (*n* = 4, 15%), Psychological Science (*n* = 2, 7.7%), Theory Into Practice (*n* = 2, 7.7%), Journal of Educational Research (*n* = 1, 3.8%), Journal of Psychoeducational Assessment (*n* = 1, 3.8%), Journal of Applied School Psychology (*n* = 1, 3.8%), Annals of the New York Academy of Sciences (*n* = 1), and Thinking Skills and Creativity (*n* = 1, 3.8%). Seven articles (27%) were published in journals indexed in the Emerging Sources Citation Index (ESCI), with the distribution as follows: Roeper Review (*n* = 3, 11.5%), Journal for the Education of the Gifted (*n* = 2, 7.7%), and Journal of Advanced Academics (*n* = 2, 7.7%). Approximately six articles (23%) appeared in non-SSCI journals, including Gifted Child Today (*n* = 3, 11.5%), Journal of Gifted Education Research (*n* = 1, 3.8%), European Journal of STEM Education (*n* = 1, 3.8%), and The American Economist (*n* = 1, 3.8%).

#### 3.1.2. Characteristics of Sampling: Age and Target Group

As depicted in Table 2, research on STEM talent identification predominantly focused on mixed-age or cross-age groups (*n* = 13, 50%), followed by studies concentrating on high school students (*n* = 9, 34.6%). However, studies targeting early identification at the preschool and elementary school settings were non-existent (0% for both). Most research concentrated on gifted students themselves, while relatively fewer studies delved into the roles and perspectives of other pivotal stakeholders, such as educators, schools, policymakers, and parents (*n* = 3, 11.5%). For instance, a study conducted in the United States explored the beliefs of high school STEM teachers concerning student talent and their instructional practices (Tofel-Grehl & Callahan, 2017), and a study from Saudi Arabia scrutinized the perspectives of mathematics teachers engaged with gifted students (AlAli et al., 2024). Furthermore, research conducted in South Korea focused on mathematically gifted high school students (Choi & Hong, 2009), whereas a study in Turkey encompassed both high schools for gifted students and the students themselves (Kanlı & Özyaprak, 2015).

### 3.2. STEM Talent Identification Strategy

#### 3.2.1. Overview of Identification Strategy

This analysis traced the evolution of STEM talent identification strategies and protocols, shifting from broad standard assessments to more targeted capability evaluations. It underscored the trends toward diversification and dynamic change in identification strategies. Initially, the identification of gifted individuals was heavily reliant on traditional intelligence tests, such as the Stanford–Binet Intelligence Scale (Terman, 1916) and the Wechsler Intelligence Scale for Children (Wechsler, 1949). These tests, which emphasized logical reasoning and verbal abilities, provided standardized data (Kanlı & Özyaprak, 2015; Ruban, 2005). However, as the importance of creativity, spatial reasoning, and non-cognitive factors, encompassing emotional and social skills, gained recognition, the limitations of these traditional methods became increasingly apparent.

In the early 21st century, identification methods began to encompass assessments of creativity and problem-solving abilities. Instruments like the Torrance Tests of Creative Thinking (Torrance, 1966) were introduced to evaluate students’ intuitive thinking and creative potential, filling the gaps left by intelligence testing (Tofel-Grehl & Callahan, 2017). Standardized exams such as the SAT and ACT, along with teacher recommendations, also gained prominence in talent identification, particularly for assessing STEM abilities (Choi & Hong, 2009; Rinn et al., 2008). Post-2010, spatial ability tests were integrated into identification systems, and nonverbal assessments like the Naglieri Nonverbal Ability Test (NNAT) were employed to identify potential in students, mitigating the limitations associated with language or cultural differences and complementing traditional intelligence tests within STEM disciplines. While these tools are crucial for identifying students with potential in engineering and technology, they demonstrated limitations in evaluating aspects such as verbal expression and creativity (Kanlı & Özyaprak, 2015).

In recent years, the identification of gifted individuals shifted toward multidimensional and comprehensive assessments, with a focus on evaluating critical thinking, practical skills, and academic performance. Performance-based assessments, such as portfolios and performance tests, along with national-level competitions (e.g., science fairs and Olympiads), became pivotal for assessing students’ critical thinking, creativity, and real-world problem-solving skills. These methods allowed students to display their talents in authentic contexts (Peters & Engerrand, 2016; Hodges et al., 2018). Despite providing a more holistic view of students’ abilities, these assessments can be costly and present challenges in ensuring consistency and fairness.

#### 3.2.2. Student-Centric Identification Approaches

The study explored student-centric identification methods for gifted students, moving beyond traditional metrics to assess a range of abilities, including intelligence, academic achievement, creativity, problem-solving, portfolio assessments, and competition participation (see Table 3). This shift reflects a transition from a unidirectional to a multidimensional approach in selecting gifted students in STEM education, aiming for a more holistic evaluation of students’ potential.

Intelligence Performance. This approach is typically assessed via standardized intelligence tests such as the Stanford–Binet and WISC, which measure cognitive skills like reasoning and processing speed to identify gifted students (Ruban, 2005). However, these tests may be biased by language and culture and overlook traits like creativity and spatial reasoning. Globally, intellectual performance assessment is tailored to fit educational priorities. The US and Turkey rely on traditional intelligence tests, with the US focusing on STEM potential and Turkey using an IQ threshold of 130 and domain-specific tests (Caropreso & White, 1994; Kanlı & Özyaprak, 2015). South Korea and Taiwan emphasize academic achievement, with South Korea prioritizing Olympiad success and Taiwan using the BCT to identify top performers (Choi, 2014; Jen & Moon, 2015). Saudi Arabia integrates innovation-focused practices and digital portfolios to track student development in STEM (AlAli et al., 2024).

Academic Performance. This approach is a key metric for identifying gifted students, typically assessed through standardized tests like the SAT and ACT, as well as GPA, aptitude tests, and essay evaluations (Rinn et al., 2008; Almarode et al., 2014; Mullet et al., 2018; Subotnik et al., 2016; Worrell & Erwin, 2011). These tools measure general academic ability but may miss specific talents, creativity, and practical skills. Globally, academic performance is evaluated differently. The US relies on SAT, PSAT, GPA (with a 3.7 threshold), and participation in advanced courses (Tofel-Grehl & Callahan, 2017; Subotnik et al., 2016). South Korea focuses on top 2% achievers in math and science (Choi & Hong, 2009), while Turkey uses traditional tests and domain-specific assessments in science and math (Kanlı & Özyaprak, 2015). Taiwan identifies gifted students through the Basic Competency Test (BCT), with scores above the 93rd percentile qualifying for gifted programs (Jen & Moon, 2015). Saudi Arabia integrates academic assessments with innovative teaching practices, using digital portfolios to document students’ learning experiences (AlAli et al., 2024).

Domain-Specific Abilities. Ability tests, such as TARC, Creative Scientific and Mathematical Ability Tests, TTCT, and Scales of Early Academic Development, are essential for evaluating creative thinking and problem-solving skills, offering a multidimensional approach to gifted identification (Caropreso & White, 1994; Kanlı & Özyaprak, 2015). However, their reliability can be culturally contingent, prompting debates on their broader applicability. Globally, domain-specific assessments are adapted to fit educational priorities. The US uses TTCT and TARC to evaluate creativity and spatial reasoning (Caropreso & White, 1994). South Korea and Turkey focus on national Olympiads and domain-specific tests, with South Korea prioritizing achievements in math, science, and IT (Choi & Hong, 2009), and Turkey using creative math and science tests (Kanlı & Özyaprak, 2015). Taiwan combines domain-specific tests with science competition results (Jen & Moon, 2015), while Saudi Arabia supports STEM learning through innovation-focused practices and assessments (AlAli et al., 2024).

Portfolio Assessment. This approach offers students the opportunity to demonstrate their abilities and potential across multiple domains through the submission of various materials, including transcripts, recommendation letters, personal statements, and evidence of giftedness (Choi, 2014). By providing a platform to display unique talents, portfolios address the limitations inherent in traditional testing methods, making them especially useful for identifying giftedness that may not be effectively reflected in standardized assessments. However, the portfolio review process involves a degree of subjectivity, and preparing these materials can be time-intensive for students. Countries adopt varied approaches to portfolio assessment, reflecting their unique educational contexts. The US and South Korea use qualitative data to supplement standardized tests, highlighting students’ personal traits and non-academic strengths (Caropreso & White, 1994; Choi, 2014). Turkey and Taiwan integrate practical activities and competition results to evaluate domain-specific potential and problem-solving skills (Kanlı & Özyaprak, 2015; Jen & Moon, 2015). Saudi Arabia innovates with digital portfolios to track student development and align with STEM education standards (AlAli et al., 2024).

National and International Competitions. Subject competitions, such as the Mathematics, Physics, and Chemistry Olympiads, are recognized globally as rigorous mechanisms for identifying gifted students, requiring extensive knowledge and resilience under pressure (Choi & Hong, 2009; Tofel-Grehl & Callahan, 2017). However, this approach, while effective for assessing specialized skills, may overemphasize performance metrics and overlook broader abilities and diverse interests. Countries tailor the use of competition results to fit local educational priorities. In the US, STEM competition achievements serve as supplementary evidence for identifying domain-specific talents (Tofel-Grehl et al., 2018). Taiwan integrates competition results with basic competency tests (BCT) to provide a comprehensive assessment of potential (Jen & Moon, 2015). South Korea prioritizes Olympiad results in core STEM fields as a direct pathway to gifted programs (Choi & Hong, 2009), while Turkey combines competition scores with domain-specific tests to evaluate potential comprehensively (Kanlı & Özyaprak, 2015).

#### 3.2.3. Teacher-And-Parent-Led Assessment Strategies

The findings of this study underscored the significance of objective evaluations conducted by teachers, principals, and parents in identifying gifted students in STEM fields. As presented in Table 4, these evaluations are multifaceted, encompassing three primary aspects: scoring, observation, and recommendation.

Teacher Observation. This method was crucial for identifying gifted students, allowing educators to directly assess their potential and performance through classroom interactions, assignments, and problem-solving abilities (Peters & Engerrand, 2016; Lee, 2016; Choi & Hong, 2009). While it captures authentic behaviors and reveals talents often missed by traditional tests (Tofel-Grehl & Callahan, 2017; Tofel-Grehl et al., 2018), it is also subjective, time-consuming, and dependent on the teacher’s training and experience (Lee, 2016). Observation methods differ significantly across countries. In the US, they integrate standardized tests and teacher evaluations to assess complex task handling. In South Korea, they link to Olympiad preparation, focusing on math and science potential. In Turkey, they emphasize hands-on activities in science and art centers to evaluate creativity and problem-solving. In Taiwan, they complement the Basic Competency Test (BCT) for a holistic assessment. In Saudi Arabia, they combine with innovative teaching practices to assess STEM learning.

Teacher/Principal Recommendations. Teacher and principal recommendations are crucial for identifying gifted students, especially when standardized tests fail to capture their full potential (Lee, 2016; Choi & Hong, 2009). Educators can identify non-academic strengths such as leadership and creativity through sustained interaction and comprehensive observation (Tofel-Grehl et al., 2018). These recommendations are particularly valuable in STEM talent identification when academic assessments fall short of reflecting a student’s holistic potential (Tofel-Grehl & Callahan, 2017). However, they may be subject to implicit biases and undermine fairness and reliability due to their subjective nature (Crabtree et al., 2019; Lee, 2016). Globally, recommendations varied in application. In the US and Taiwan, they integrate standardized test scores, classroom performance, and competition results for a holistic assessment (Caropreso & White, 1994; Jen & Moon, 2015). South Korea and Saudi Arabia focus on academic achievements, Olympiad performance, and innovative teaching practices, often endorsed by school leaders (Choi & Hong, 2009). Turkey combines academic ability with performance in science and art centers.

Parent and Teacher Ratings. This method provides a multifaceted perspective in the identification process, offering supplementary information about students’ potential based on observations of their behaviors across home and school settings. Caropreso and White (1994) proposed that gifted subjects are identified through creativity tests, parent and teacher ratings, and standardized intelligence tests. Parents identify students’ potential outside of school through parent questionnaires, while teachers contribute a more comprehensive assessment of students’ academic and social performance. This multidimensional rating approach addresses the limitations of single assessment tools, allowing for a more holistic evaluation of students’ potential for exceptional achievement in STEM.

#### 3.2.4. Emerging Trends in Identification Practice

This study identified several emerging trends in the practice of STEM talent identification, including technology integration, data-driven decision-making, and the application of multiple intelligences theory. First, technology integration is transforming gifted identification, particularly through the use of digital portfolios powered by artificial intelligence and big data (Crabtree et al., 2019). This integration allows schools to analyze individual student data with greater precision to identify those with high potential for exceptional achievement in STEM. Second, data-driven decision-making provides a more scientific basis for identifying gifted students by analyzing academic performance, classroom engagement, and personalized learning pathways (Andersen, 2014). Third, the integration of multiple intelligences theory, which posits that giftedness extends beyond linguistic and logical skills to include spatial, musical, and bodily-kinesthetic intelligences (Kell et al., 2013b), has significantly broadened the scope of talent identification methods. This theoretical framework facilitates a more nuanced approach to recognizing and fostering students’ diverse abilities, thereby supporting talent development across multiple domains (Guo & Woulfin, 2016). These trends contribute to a more diversified and adaptable framework for gifted identification, allowing the process to meet the dynamic and evolving demands of contemporary education and societal advancement.

### 3.3. Key Findings on STEM Talent Identification

This study’s findings emphasize the critical roles of creativity (Choi, 2014), problem-solving skills (Sen et al., 2021), cognitive reasoning (Caropreso & White, 1994), and spatial ability (Andersen, 2014) in the identification of gifted students in STEM fields. These competencies are essential indicators for recognizing students with high potential in STEM disciplines. Moreover, understanding the development of these competencies is further complicated by the influence of a multitude of societal and educational factors, including ethnicity and cultural diversity (Caropreso & White, 1994), socio-economic status (Crabtree et al., 2019), gender differences (Almarode et al., 2014), and parents’ educational background and familiarity with STEM (Peters & Engerrand, 2016).

#### 3.3.1. Creativity and Problem-Solving Skills

While creativity and problem-solving abilities are paramount in identifying talented individuals, the effectiveness of current assessment methods remains debated. Choi (2014) noted a significant shift in the admission protocols of specialized Korean STEM schools, which now evaluate creativity and psychological attributes alongside academic excellence. This reflects a commitment to nurturing future STEM leaders through a dynamic, inquiry-driven curriculum that values creativity, contrasting with the more achievement-focused approach of Science High Schools. However, the nuances of creative thinking may not be fully captured by existing assessment tools. The Science Academies’ admission process, which includes assessments of creativity and personal interviews, aims to identify students with potential to excel in scientific and mathematical disciplines, moving away from an over-reliance on academic competition outcomes. Sen et al. (2021) emphasized the robust problem-solving skills of gifted and talented students, as demonstrated by their proactive engagement in addressing complex problems within STEM initiatives. These students effectively devised, implemented, and evaluated the effectiveness of their solutions, showcasing their proficient computational thinking. However, current educational frameworks may be insufficient in fostering and recognizing the full spectrum of problem-solving skills necessary for innovation in STEM. The research concluded that engineering design process STEM activities provide an invaluable framework for identifying and refining the problem-solving abilities of gifted and talented students, while also raising concerns about the potential for overemphasis on structured problem-solving at the expense of open-ended creativity.

#### 3.3.2. Cognitive Reasoning and Spatial Ability

Cognitive reasoning and spatial ability also play significant roles in STEM talent identification. Caropreso and White (1994) conducted a study using the Test of Analogical Reasoning in Children (TARC) to meticulously measure young children’s capacity to solve geometric analogy problems, a skill that served as a strong indicator of their cognitive reasoning capabilities. However, the generalizability of these findings to diverse populations and different educational contexts must be considered. In this study, gifted children consistently demonstrated superior performance compared to their non-identified peers, thereby highlighting a significant disparity in analogical reasoning abilities. Furthermore, Kell et al. (2013b) found that spatial ability, along with mathematical and verbal reasoning as measured by the SAT, significantly predicted creativity and innovation among intellectually talented individuals. Spatial ability uniquely contributes to the cultivation of creativity and should be considered in educational and psychological assessments. However, the extent to which spatial ability can be developed and the most effective methods for doing so remain open questions. Building on this, Andersen (2014) emphasized that visual–spatial ability is a critical yet often overlooked component of intelligence for success in STEM fields. Despite its predictive validity for achievements in science, technology, engineering, and mathematics, this ability is rarely measured in gifted education programs. The traditional focus on quantitative and verbal reasoning has led to the underidentification of high-spatial gifted students, potentially contributing to a national shortage of domestically produced scientists and engineers. The article emphasizes the need to incorporate spatial ability assessments in identifying and nurturing STEM talent. However, this creates the challenge of developing assessments that can accurately measure spatial ability without cultural or gender biases, which is a crucial step toward addressing the issues highlighted by these studies.

#### 3.3.3. Societal and Educational Influences

The process of talent identification and development in STEM fields is influenced by a complex array of societal and educational factors. Caropreso and White (1994) highlighted that ethnicity and cultural diversity significantly impact analogical reasoning among the gifted, suggesting that cognitive abilities are influenced by cultural backgrounds. This underscores the necessity to consider cultural diversity when nurturing talent within STEM. Simultaneously, the educational background and STEM literacy of parents, as Peters and Engerrand (2016) noted, substantially shape the developmental trajectories of gifted students, indicating that a supportive home environment can significantly enhance a child’s interest and proficiency in these areas. Furthermore, socio-economic status (SES), as observed by Crabtree et al. (2019), plays a critical role in the recognition of talent, with low-SES students often being underrepresented in gifted programs—a disparity that Andersen (2014) emphasizes the need to address urgently. Moreover, despite possessing equal potential, gender biases and societal expectations, as documented by Rinn et al. (2008), Almarode et al. (2014) and Green and Sanderson (2018), contribute to lower self-efficacy and participation among female students in STEM. Collectively, these findings highlight the importance of implementing a comprehensive and equitable approach to talent identification and development. Such an approach must address and mitigate the effects of these diverse influences to ensure that all individuals, regardless of their background, are afforded the opportunity to excel in the fields of science, technology, engineering, and mathematics.

The studies collectively illustrate the importance of assessing a broad spectrum of skills beyond traditional academic metrics when identifying and nurturing the next generation of STEM leaders. They advocate for a more holistic and multifaceted approach to talent identification, which not only recognizes but also fosters the creative and problem-solving capabilities that are vital for success in the STEM fields. The critical analysis of these studies reveals that while they provide valuable insights, there is a need for continuous refinement of assessment tools and educational practices to ensure that the identification and development of STEM talent is equitable and effective.

## 4. Discussion

The present review observes a discernible trajectory in STEM talent identification, seemingly moving from a sole reliance on standardized intelligence testing toward multidimensional, context-sensitive approaches. It is important to note that while this trend aligns with established theoretical frameworks (e.g., Renzulli, 1978, 2005), the current synthesis did not quantitatively model publication year as a variable. Consequently, this characterization of evolution is derived from a qualitative synthesis of the literature’s content and emphasis, rather than a quantitative frequency analysis across decades.

First, this systematic review indicated that STEM talent identification research has been predominantly focused on the U.S. context, with a peak in publications in 2018 followed by a decline. However, this geographic concentration constitutes a significant Western-centric bias. Since the Cold War, the United States had prioritized technological advancement and STEM talent cultivation, resulting in more research output compared to other countries. While nations such as the United Kingdom, France, South Korea, and China also emphasized STEM talent development, fewer studies were published in English, with many appearing in Korean, Turkish, and Chinese (Han, 2007; Jeong & Yoon, 2012; Ülger & Çepni, 2020; Shi, 2024). Consequently, the findings reported here primarily reflect identification practices within Anglo-American education systems and should not be generalized to non-Western contexts without further validation. The decline in STEM identification studies after 2019 may have partly reflected a proposed shift towards STEM development programs (Assouline et al., 2023; Stoeger et al., 2024), such as university–school STEM partnerships and digital labs to enhance engineering skills. Nevertheless, this proposed shift also risks diverting attention and resources away from the fundamental challenge of early identification.

Furthermore, among the twenty-six reviewed articles, empirical studies outnumbered reviews, focusing mainly on high school students, while research on younger students was limited. High school is recognized as a pivotal phase for fostering scientific literacy and shaping career trajectories. Yet, this emphasis on adolescence appears incongruent with developmental frameworks, such as the Talent Development Megamodel (TDMM), which posits that domain-specific competencies are progressively refined from early childhood into adulthood. In the 21st century, driven by advancements in artificial intelligence and technology, early identification and cultivation of STEM-related potential were critical for fostering innovative talent and represented important future research directions. The current neglect of early childhood in the literature thus represents a critical gap that future studies must address.

Second, the study highlighted a diverse and dynamic shift trajectory in STEM talent identification methods, transitioning from a holistic to an individualized perspective. These methods included measures of intelligence, academic performance, creativity, problem-solving, and spatial skills, along with portfolio reviews, disciplinary competition recognitions, gifted programs, and teacher and parent evaluations. This diversification reflected a growing awareness that a single test could not fully capture the range of STEM talents, as noted by scholars such as Sternberg (2010) and Renzulli (2005), who emphasized that traditional assessments might overlook students with unconventional or latent talents, particularly those that emerged in practical applications. However, the field currently lacks consensus on how these disparate measures should be weighted or integrated, leaving practitioners without clear guidance on implementing multidimensional identification.

Accordingly, teacher observations, intelligence tests, and ability assessments collectively contribute to a more comprehensive, multidimensional perspective in talent identification. Nevertheless, a structural tension persists between standardized measures and authentic assessments. Intelligence tests offer psychometric rigor and scalability but often fail to capture the dynamic, context-dependent nature of STEM problem-solving. Conversely, digital portfolios provide rich, ecologically valid evidence of creativity and persistence, yet they suffer from subjective scoring and high resource demands. The field’s failure to reconcile this trade-off between psychometric validity and practical feasibility perpetuates a core barrier to equitable implementation. Future research must move beyond advocating for one approach over the other and instead investigate hybrid models that leverage the strengths of both. Building on this need for diversification, the integration of digital portfolios aligns with the growing importance of technology in education, facilitating personalized learning pathways and assessment methods. However, empirical evidence validating the reliability and fairness of these tools remains nascent. This dynamic approach reflects an understanding of STEM talent as a developmental process rather than a fixed attribute, aligning with Dweck’s (2006) assertion that talent can be cultivated through appropriate educational interventions. Yet, translating this theoretical flexibility into scalable, system-wide practice remains a significant challenge.

The 21st-century STEM landscape, with its complex challenges, required an equally complex system of talent identification. The field sought individuals who could integrate interdisciplinary knowledge, engage in cross-domain collaboration, and demonstrate creative problem-solving skills, necessitating a multifaceted identification approach. While the rationale for this multifaceted approach is conceptually sound, its operationalization remains fragmented across different educational jurisdictions. Thus, the dynamic evolution of STEM talent identification was not simply a reflection of educational trends but a necessary adaptation to meet the demands of the modern STEM field. The criteria for identifying STEM talent broadened, now including creativity, spatial reasoning, and critical thinking alongside intelligence. Renzulli (2005) emphasized that creativity and academic ability should be considered complementary, underscoring the need to recognize students with strong creative abilities in the identification process. Spatial reasoning, another critical dimension, had become increasingly essential, particularly in fields like engineering, architecture, physics, and computer science, where prominent levels of spatial cognition were required. Notwithstanding its importance, spatial ability remains underutilized in mainstream frameworks, suggesting a disconnect between research evidence and policy practice. Studies suggested that enhancing spatial abilities could significantly improve students’ long-term performance in STEM, leading more identification systems to include spatial reasoning as a core criterion. Finally, the inclusion of critical thinking strengthened the multidimensionality of identification systems, as critical thinking was essential for adapting to the rapidly changing technological and social landscape.

Third, this study underscored that STEM talent identification was influenced not only by core competencies like cognitive reasoning, creativity, and spatial ability but also by broader social and educational influences, including socioeconomic status, cultural background, and gender expectations. Key competencies directly impacted STEM talent recognition, aligning with STEM education frameworks. Spatial ability, highlighted by Andersen (2014), uniquely predicted creative outcomes. Despite its strong predictive validity, spatial ability is often marginalized in favor of more traditional, verbally loaded metrics. Since 2014, scholars have advocated for its formal integration, calling for more empirical research. Other researchers, such as Xu and Yuan (2011), expanded understanding of spatial abilities. Korean scholars suggested spatial ability, as a non-verbal skill, could serve as an assessment tool less affected by cultural, linguistic, and socioeconomic biases (Jeong & Yoon, 2012). However, access to spatial skill development itself is often stratified by socioeconomic status, complicating claims of cultural neutrality. Future identification strategies should incorporate standardized measures, assessment criteria, and curricula to support spatial skill development.

Moreover, factors like socioeconomic status, cultural background, teacher and parent support, and gender expectations indirectly impacted STEM talent identification. Children from high-SES families had access to more resources, which could enhance standardized test and ability assessment outcomes. Cultural variations significantly influenced definitions and expectations of giftedness. Teachers and parents played essential roles in recognizing STEM talent, though teacher subjectivity could introduce bias. This reliance on subjective nominations, while valuable, risks reinforcing existing privilege unless accompanied by objective safeguards. Parental support enhanced academic motivation and STEM achievement, though not all parents had resources to fully support development. Gender expectations also shaped STEM talent identification, as society often held different expectations for males and females in STEM fields. These gendered perceptions continue to suppress female representation in certain STEM domains. Future systems should address gender equity, mitigate implicit biases, and implement support for female students in STEM. Equitable STEM talent identification would require integrating socioeconomic status, cultural background, teacher and parent support, and gender expectations. Not only would this approach enhance identification accuracy, but it would also reduce inequalities. Policymakers and researchers could advance these goals by creating diverse assessment tools that captured characteristics of students from varied backgrounds, implementing socioeconomic support programs, strengthening parent–school collaboration, and designing gender-neutral frameworks. Such structural adjustments are imperative if identification systems are to move beyond merely documenting disparities to actively dismantling them.

Beyond these theoretical and social complexities, the ultimate utility of this body of research hinges on its practical implementation. While this review synthesizes a broad spectrum of identification approaches, a critical challenge remains the translation of these theoretical constructs into actionable protocols for educators. The current evidence base, characterized by methodological diversity but lacking consensus on measurement weights, does not yet support a universally validated identification protocol. To bridge this gap, future research must prioritize the development of predictive models to determine which specific indicators or combinations thereof, most robustly forecast STEM talent across diverse contexts. Crucially, the field must move beyond documenting disparities to constructing practical frameworks that guide educators in selecting, weighting, and implementing these multifaceted tools. Such frameworks should offer clear decision-making pathways for practitioners, ensuring that the proposed shift toward multidimensional identification results in tangible improvements in equity and accuracy. Only by operationalizing these insights can the field transition from conceptual aspiration to systemic reform, thereby transforming the identification process into a catalyst for cultivating diverse STEM talent.

### Limitations and Future Directions

This study acknowledges several limitations that influence the scope and applicability of its findings. We excluded 79 non-English articles during literature screening primarily to guarantee consistent coding standards and stable inter-rater reliability among the review team, yet this exclusive reliance on English-language literature inevitably creates prominent Anglo-American/Western-centric linguistic bias. Future research must critically address this linguistic hegemony. A multilingual systematic review approach, incorporating non-English sources from diverse non-Western regions, such as Chinese academic literature indexed in the China National Knowledge Infrastructure (CNKI), is essential to enrich the global discourse on STEM talent identification.

Future research on STEM talent identification must critically interrogate current practices and their potential long-term impacts, particularly regarding educational disparities and opportunity gaps. Longitudinal studies are essential to evaluate the efficacy of existing strategies, with a focus on traditionally marginalized student groups, such as those from low-income backgrounds (Crabtree et al., 2019), ethnic minorities (Maker et al., 2024), and females (Jen & Moon, 2015), whose STEM potential is frequently overlooked. Current literature too often documents these gaps without offering actionable pathways to close them. Cross-cultural comparisons should also be expanded to explore the diversity and variation in STEM talent identification practices across different cultural contexts, moving beyond Western-centric paradigms to understand how various educational systems and cultural values shape the definition and implementation of STEM talent identification (Lee, 2016; Tofel-Grehl et al., 2018). This comparative lens is necessary to challenge the universality of existing global educational standards.

Furthermore, a related caveat is that the current synthesis is limited in its capacity to chart methodological trends over time. Future meta-analytic research should address this by incorporating publication year as a key moderator variable. Specifically, researchers should adopt the practice of coding identification methods by distinct publication periods (e.g., by decade) and reporting their frequencies over time. Such quantitative trend analysis is essential to empirically substantiate whether a true evolution in assessment practices is occurring and to correlate these changes with specific historical or policy-driven contexts.

Moreover, the integration of emerging technologies, such as artificial intelligence and big data analytics, into STEM talent identification processes requires rigorous scrutiny. While these technologies offer promising opportunities for innovation, they may also introduce new biases or exacerbate existing inequities. Future research must critically assess the utility and fairness of these technologies to ensure they equitably serve all students. This evaluation will provide valuable insights for educators to refine assessment tools and methods and inform policy decisions aimed at promoting educational equity and innovation. Ultimately, a more inclusive and diverse approach to STEM education is imperative for ensuring that resources and opportunities are accessible to all students, regardless of their backgrounds. This will lead to a fairer and more effective STEM education system that nurtures the development of future scientists, engineers, and innovators.

## 5. Conclusions

This systematic review synthesizes the extant literature on STEM talent identification in K-12 education settings. The findings reveal key characteristics and trends in STEM talent identification research, illustrating a shift from a reliance on standardized testing and intelligence performance toward a more diversified, multidimensional approach. This evolution encompasses domain-specific assessments, such as creativity, problem-solving skills, and spatial ability, alongside portfolio evaluations, national and international competitions, teacher or parent observations and recommendations. These developments reflect notable advances in educational research and a nuanced appreciation of student potential. However, existing methods still have limitations, underscoring the need for further empirical research and practical models targeting the early identification of talent within specific STEM domains. Furthermore, the findings highlight the indirect influence of social and educational factors on STEM talent identification, suggesting that future identification systems must comprehensively consider these elements to ensure fairness. By clarifying prevailing practices and evidentiary gaps, this review offers actionable insights for policymakers, school administrators, and educators within comparable educational contexts, aiding in the optimization of strategies for identifying STEM talent. Moreover, these findings contribute to a stronger theoretical foundation for STEM talent identification and provide practical guidance for fostering inclusive and diverse STEM education environments that support the development of future scientists, engineers, and innovators.

## Figures and Tables

**Table 1:** Coding Rules for Reviewed Articles.

Content	Description
General Features	Country (in text)
	Year of publication (in text)
	Types of publication: empirical study = 1, review article = 2, others = 3
	Journals (in text) and indexes: SSCI = 1, SCI = 2, others = 3
Content Features	Methodology: quantitative = 1, qualitative = 2, mix = 3
	Sample: N, male = 1, female = 2
	Participants: gifted students = 1, non-gifted students = 2, both = 3, educators, schools, policymakers, parents, etc. = 4
	Educational level categorization: kindergarten = 1, primary = 2, secondary = 3, middle = 4, high = 5, mixed/cross-age group = 6
	Open coded strategy and content analysis for the instruments, key findings, limitations, and implications of studies of STEM talent identification

**Table 2:** Summary of the Demographic Information of Participants in the Reviewed Articles.

	N (%)
**Educational Level**	
Kindergarten	0 (0)
Primary school	0 (0)
Secondary school	1 (3.8)
Middle school	3 (11.5)
High school	9 (34.6)
Mixed/cross-age group	13 (50)
**Target group**	
Gifted students	19 (73.1)
Non-gifted students	0 (0)
Both gifted and non-gifted students	3 (11.5)
Educator, schools, parents, etc.	3 (11.5)
Mixed group1	1 (3.8)

**Table 3:** Student-Centric STEM Talent Identification Method and Instruction.

Identification Method	Instrument and Description	Standard	References
Intelligence Performance			
Intelligence Tests	Stanford–Binet Intelligence Scale Wechsler Intelligence Scale for Children and Adults (WISC) Cattell Culture-Fair Intelligence Test Otis-Beta Quick Scoring Mental Ability Test	A typical cutoff IQ score is 130	Ruban (2005) Kanlı and Özyaprak (2015) Hodges et al. (2018)
Nonverbal Ability Tests	Naglieri Nonverbal Ability Test	A standard score of 130 or above or a percentile rank of 98 or above	Peters and Engerrand (2016)
Performance-Type Tests	Goodenough Draw-a-Man Test Torrance Tests of Creative Thinking	Scoring within the top 5–10%	Kanlı and Özyaprak (2015)
Academic Performance			
Standardized Tests	Scholastic Aptitude Test (SAT) (include Critical Reading, Math, and Writing)	Scored at or above the 95th percentile for the combined SAT	Almarode et al. (2014)
		At age 13, scores of at least 700 on SAT-M or 630 on SAT-V, placing them in the top 1 in 10,000 in mathematical or verbal reasoning.	Kell et al. (2013a)
	American College Test (ACT)	A score of 28 or higher	Mullet et al. (2018)
	Basic Competence Test (BCT)	Students who scored above the 93rd percentile on the BCT standardized national test in Taiwan	Jen and Moon (2015) Subotnik et al. (2016)
	Aptitude Tests	Students identified as potential candidates took an aptitude test, and the top 30 based on their scores were selected for the gifted program	Kanlı and Özyaprak (2015) Jen and Moon (2015) Crabtree et al. (2019)
	Illinois Standards Achievement Test (ISAT)	Math scores range from 120 to 341 (or 410, depending on grade) Reading scores range from 120 to 329 (or 279, depending on grade)	(Olszewski-Kubilius, 2023)
Grade Point Average	GPA	A GPA of at least 3.75 on a 4.00 scale	Mullet et al. (2018)
Creativity and Problem-Solving Skills			
Test of Analogical Reasoning in Children (TARC)	An individually administered tool, children’s ability to solve geometric analogy problems was measured.	Gifted students typically score within the top 10–20% of their age group, demonstrating exceptional performance.	Caropreso and White (1994)
Torrance Tests of Creative Thinking (TTCT)	Commonly used to evaluate students’ creative thinking abilities, including fluency, originality, and elaboration.	Scoring above 130 is typically considered to demonstrate high potential for exceptional achievement in STEM; scoring in the top 2–3% (98th percentile or above) or top 10% is considered exceptional and suitable for further attention or inclusion in gifted education programs.	Caropreso and White (1994)
Alternative Creativity Tests	Tests of Mathematical Talent	The top 5–10%, or at the 98th percentile or above	Kanlı and Özyaprak (2015)
	Creative Scientific Ability Test		
	Creative Mathematical Ability Test		
	Scales of Early Academic Development		
Spatial Ability	Spatial Ability Test	The top 5–10%, or at the 98th percentile or above	Kell et al. (2013b) Andersen (2014) Hodges et al. (2018)
Portfolio Assessments	Transcripts, letters of recommendation, a personal statement, and authentication of giftedness	Applicants submit a collection of documents, including school records, recommendation letters, personal statements, and an “authentication of giftedness.” Additionally, students submit personal statements and evidence of achievements (e.g., research projects, science competitions) to demonstrate their giftedness in STEM fields. The latter is intended to highlight a student’s exceptional abilities in STEM without relying on competition results or participation in gifted programs.	Worrell and Erwin (2011) Hodges et al. (2018)
National and International Competitions	Winners of Various National Olympiads	This method prioritizes winners of various national Olympiads in subjects like Mathematics, Science, or Information Technology, admitting up to 36 medal-winning students through this route.	Choi and Hong (2009)
	National Science Fair Achievements	Students who had won at least one National Science Fair prize during middle school were eligible to apply.	Jen and Moon (2015)

**Table 4:** Teacher-and-Parent-Led STEM Talent Identification Method and Instruction.

Identification Method	Instrument and Description	Reference
Observation	Curriculum-based Assessments: Systematic evaluations of academic work to monitor student performance over time. Behavioral Observations: Monitoring of classroom engagement, creativity, and problem-solving abilities.	Ruban (2005) Worrell and Erwin (2011) Peters and Engerrand (2016) Maker et al. (2024)
Recommendation	Teacher Recommendations: Subjective evaluations and nominations of students based on interest, creativity, and performance in STEM activities.	Choi and Hong (2009) Choi (2014) Tofel-Grehl and Callahan (2017) Tofel-Grehl et al. (2018) Hodges et al. (2018) Crabtree et al. (2019)
Rating	Parental and Teacher Input: Feedback on a child’s behavior and cognitive skills from both home and school environments.	Caropreso and White (1994)

## Data Availability

The data presented in this study are available on reasonable request from the corresponding author.

## Supplementary Materials

The following supporting information can be downloaded at: https://www.mdpi.com/article/10.3390/jintelligence14080153/s1, S1: PRISMA 2020 checklist.
